# Development of an Antioxidant Phytoextract of *Lantana grisebachii* with Lymphoprotective Activity against *In Vitro* Arsenic Toxicity

**DOI:** 10.1155/2014/416761

**Published:** 2014-06-05

**Authors:** Elio A. Soria, Patricia L. Quiroga, Claudia Albrecht, Sabina I. Ramos Elizagaray, Juan J. Cantero, Guillermina A. Bongiovanni

**Affiliations:** ^1^Facultad de Ciencias Médicas, Universidad Nacional de Córdoba, INICSA-CONICET/UNC, Enrique Barros S/N, 5014 Córdoba, Argentina; ^2^Consejo Interuniversitario Nacional, Pacheco de Melo 2084, 1126 Ciudad Autónoma de Buenos Aires, Argentina; ^3^Facultad de Agronomía y Veterinaria, Universidad Nacional de Río Cuarto, IMBIV-CONICET/UNC, Ruta 36 Km 601, 5804 Río Cuarto, Argentina; ^4^Facultad de Ciencias Agrarias, Universidad Nacional del Comahue, PROBIEN-CONICET/UNCO, CP 8300, Neuquén, 1400 Buenos Aires, Argentina

## Abstract

Phytochemicals have been presumed to possess prophylactic and curative properties in several pathologies, such as arsenic- (As-) induced immunosuppression. Our aim was to discover a lymphoprotective extract from *Lantana grisebachii* Stuck. (Verbenaceae) (LG). We assessed its bioactivity and chemical composition using cell-based assays. Fractions produced from a hexane extract acutely induced nitrite formation in T-activated cell cultures (*P* < 0.0001). Water extraction released a fraction lacking nitrite inducing activity in both lymphocyte types. Aqueous LG was found to be safe in proliferated and proliferating cells. The infusion-derived extract presented better antioxidant capacity in proportion to phenolic amount in lymphocytes (infusive LG-1i at 100 **μ**g/mL), which protected them against *in vitro* As-induced lymphotoxicity (*P* < 0.0001). This infusive LG phytoextract contained 10.23 ± 0.43 mg/g of phenolics, with 58.46% being flavonoids. Among the phenolics, the only predominant compound was 0.723 mg of chlorogenic acid per gram of dry plant, in addition to 10 unknown minor compounds. A fatty acid profile was assessed. It contained one-third of saturated fatty acids, one-third of **ω**9, followed by **ω**6 (~24%) and **ω**3 (~4%), and scarce **ω**7. Summing up, *L. grisebachii* was a source of bioactive and lymphoprotective compounds, which could counteract As-toxicity. This supports its phytomedical use and research in order to reduce As-related dysfunctions.

## 1. Introduction

Many Argentinean plant species have been proposed as sources of bioactive compounds that might be used to prevent and treat several human health pathologies [1]. Among these compounds, phenolics are the main candidates for this biomedical potential, given their antioxidant and multitarget effects. These processes involve xenohormesis, which is an organic enhancement of cellular resistance against oxidative stress acquired by consuming plant-synthesized compounds [2]. Oxidative stress underlies numerous chronic dysfunctions by triggering a redox imbalance with free radical overproduction (reactive species of O, N, or S) and impairment of antioxidant defence [3]. Reactive species can be generated endogenously by cellular mechanisms or be induced exogenously by environmental agents, such as pollutants (e.g., arsenic, pesticides, etc.) [4].

The immune system involves a complex integration of biological defences intended to protect an organism against numerous pathogens, with B and T lymphocytes being the crucial cells involved [5]. Given that the immune system is one of the main targets affected by environmental oxidants (secondary immunosuppression), immune recovery might be achieved by implementing certain bioactive phytochemicals with immunoxenohormetic activity [6]. Accordingly, antioxidants could be used as chemopreventive immunoregulatory agents against chemically induced stress. A classic example is chronic hydroarsenicism or arsenicosis: a multisystem syndrome due to prolonged arsenic intake from drinking water. Worldwide, it presents high sanitary impact. Arsenic impairs the redox response of cells leading to oxidative damage by bottom-up cytotoxicity [7], with immunotoxic effects impairing cellular and antibody responses [8]. Furthermore, it exacerbates the inflammatory response [9].

In this area, phytopharmacological bioprospecting in Argentina is promising. Several potentially beneficial species inhabit in the mountainous region of central Argentina.* Lantana grisebachii* Stuck. ex Seckt. var.* grisebachii* (Verbenaceae) was selected after ethnopharmacological and experimental studies. Infusions of the aerial parts of this plant are traditional gastrointestinal stimulants, as they improve toxin clearance and possess antipyretic and antimicrobial activities [10]. All of this suggests an immunoactive potential. In addition, it exhibits antioxidant activity in food and prevents* in vitro* arsenic nephrotoxicity [11]. From these studies, this species has been proposed as sources of polyphenols [12]. Among these bioactive molecules, phenolic acids and flavonoids are the most extensive groups with antioxidant properties, whose acquisition depends on genetic, environmental, and technical variables [13].

The aim of this study was to develop an anti-As extract from* L. grisebachii *(LG), through establishing its bioactivity with cell-based assays and then its chemical composition. Specific objectives were to assess optimal extraction method, redox safety, and antioxidant and lymphoprotective effects.

## 2. Materials and Methods

### 2.1. Plant Processing

Argentinean* Lantana grisebachii* (LG) of the Chaquenian phytogeographic region [14] was collected in summer (GPS coordinates: −31.28, −64.44), after obtaining government consent by MinCyT-Cba. Specimens were deposited in the RIOC Herbarium (UNRC, Argentina). One gram of pulverized, air-dried aerial parts was extracted in the dark at room temperature under constant shaking with 4 mL of hexane (LG-24h: hexanic extraction), water (LG-24m: 24 h aqueous maceration), or water initially at 95°C (LG-1i: 1 h aqueous infusion). Then, extracts were recovered from the supernatants by filtration (0.45 *μ*m HAWG04756 filters, Millipore, Brazil) and 24 h lyophilisation to be later dissolved in 50% dimethylsulfoxide (Sigma, USA).

### 2.2. Animal Care and Cell Culture

Wistar rats (*n* ≥ 6) of both sexes were cared for according to US ethical guidelines and bred under standard laboratory conditions with* ad libitum* potable <0.01 mg As/L water (Aguas Cordobesas SA, Argentina) and commercial food (fatty acid profile: 14 : 0 (1.3%), 14 : 1 *ω*9 (1.8%), 16 : 0 (21%), 16 : 1 *ω*7 (0.6%), 18 : 0 (26%), 18 : 1 *ω*9 (11.5%), 18 : 2 *ω*6 (23.8%), 18 : 3 *ω*3 (2.1%), 20 : 1 *ω*9 (0.5%), 20 : 2 *ω*6 (1.3%), 20 : 4 *ω*6 (0.2%), 20 : 5 *ω*3 (6.7%), and 22 : 1 *ω*9 (0.2%)) (Cargill SACI, Argentina). After that, splenocytes were obtained by mechanical dispersion and chemical haemolysis of the spleens, and they were cultured at 37°C in a 5% CO_2_ atmosphere in a RPMI-1640 medium with 10% foetal bovine serum, 100 *μ*M ciprofloxacin, and 50 *μ*M 2-mercaptoethanol (Sigma, USA). Then,* ex vivo *mitogen-induced activation (EVMIA) was achieved by treating 1000 cells/*μ*L with 5 *μ*g/mL of concanavalin A or lipopolysaccharide for 72 hours, to induce T-or B-activated splenocytes, respectively. All outcomes were standardized by results in unstimulated cell cultures, with a 72 h limit proliferation.

### 2.3. Experimental Design

#### 2.3.1. Identification of Safe Fractions

First, the effects of polar and nonpolar LG fractions (200 *μ*g/mL, 2 h) were compared in already stimulated splenocytes (after EVMIA) to discard intrinsic extract toxicity. After the polar fraction was shown to be safe, aqueous extracts were studied in dividing cells (during all EVMIA; 100 *μ*g/mL, 3 d). Also, given that* in vivo* insults could affect responses, two cell sources were used: C (control group) and As (2-month orally exposed rats to 5 mg/Kg/d of As from NaAsO_2_, Anedra Lab, Argentina). These conditions were an accepted rat model of arsenicosis [15], with nitrites being oxidative (61% correlated to free radicals) and inflammatory biomarkers.

#### 2.3.2. Identification of an Efficient Extract

Redox efficiency (*see below*) of the safe aqueous fractions (100 *μ*g/mL, 3 d) was tested during EVMIA.

#### 2.3.3. Assessment of Direct* In Vitro* Protective Activity

The most efficient and safe phytoextract was assayed during EVMIA in cells exposed to 0–7.5 *μ*g/mL of As, with these conditions triggering high toxicity and allowing screening protective agents [16].

### 2.4. Biological Tests

#### 2.4.1. Cellular Viability

Since the Trypan blue exclusion test is not a sufficient determination of viability, a resazurin-based assay was employed. Viable cells were stained with resazurin (0.05 mg/mL in culture medium, 6–12 h; TOX-8 kit, Sigma-Aldrich, USA) [17]. Then, viability was calculated as the percentage of absorbance at 600 nm with respect to control (C = 100%). Absorbance readings were performed with a GloMax-Multi microplate multimode reader (Promega Corp., USA).

#### 2.4.2. Cellular Nitrites

Nitrites, used as nitrosative stress markers, were assayed by the Griess reaction [18], with reactants purchased by Wiener Lab (Argentina). Cell suspensions reacted with equal volumes of 0.1% naphthylethylenediamine dihydrochloride and 1% sulphanilamide in 0.1 N HCl (room temperature, 15 min). Percentages were calculated from a standard sodium nitrite curve (at 550 nm).

#### 2.4.3. Free Radical Activity

Radicals oxidized an ethanolic 16 mM N,N,N′,N′-tetramethyl-p-phenylenediamine-1,4-dihydrochloride solution (Sigma, USA) to be read at 540 nm [3]. Equal volumes of cell sample and solution reacted for 30 min in an oxygen-free environment at room temperature. Percentages, with respect to control, were used to calculate redox efficiency as the quotient of radical activity (%) over the extract phenolic content (%).

### 2.5. Phytochemistry

#### 2.5.1. Total Phenolics

A solution was created with 25 *μ*L of extract, 25 *μ*L of 2N Folin-Ciocalteau (Anedra, Argentina), and 150 *μ*L of water, and then 50 *μ*L of saturated sodium bicarbonate solution was added. After 30 min of incubation at 37°C in the dark, absorbance was recorded at 750 nm [19]. A standard curve was used to calculate mg equivalents of gallic acid per gram of dry extract (mg/g). Gallic acid was from Riedel-de-Haën (China).

#### 2.5.2. Total Flavonoids

Flavones and flavonols were determined as follows [20]: 50 *μ*L of extract was incubated for 30 min at room temperature with 150 *μ*L of ethanol (96%) (Cicarelli, Argentina), 10 *μ*L of aluminium chloride (10%), 10 *μ*L of potassium acetate (1 M) (Anedra, Argentina), and 150 *μ*L of water. Results were calculated at 415 nm as mg equivalents of quercetin dihydrate per gram of dry extract (mg/g) using a standard curve (Fluka, UK).

#### 2.5.3. Phenolic Analysis

Phenolics were analyzed by high performance liquid chromatography with diode array detection with a HPLC-DAD Agilent Technologies 1200 Series system equipped with Agilent G1312B SL Binary gradient pump, Agilent G1379 B solvent degasser, Agilent G1367 D SL + WP autosampler, and Agilent G1315 C Starlight DAD (ISIDSA, UNC). Separation was achieved on a LUNA reversed-phase C18 column (5 *μ*m, 250 mm × 4.60 mm i.d.; Phenomenex, USA), set at 35°C using an Agilent G1316 B column heater module. The mobile phase was 0.5% formic acid (Fluka, Germany) in ultrapure water (<5 *μ*gL-1 TOC; Sartorius, Germany) (vv-1, solvent A) and 0.5% formic acid in methanol (Baker, Mex.) (vv-1, solvent B). It began at 20%, rising to 50% B in a period of 3 min, maintained for 5 min, followed by a second increase to 70% B in the course of 7 min, maintained for 5 min, and a third increase to 80% B in 1 min, maintained for 9 min, remaining at this last concentration for 10 min before being run. The flow rate was 0.4 mL/min, injecting 40 *μ*L into the column. DAD was set at 280, 320, and 350 nm as preferred wavelengths and the UV-Vis spectra were 200–600 nm. Standards were ferulic acid and caffeic acids (Extrasynthese, France), naringin, kaempferol, and p-coumaric acid (Fluka, UK), and chlorogenic acid, naringenin, myricetin,* trans*-resveratrol, and rutin (Sigma-Aldrich, Germany).

#### 2.5.4. Fatty Acid Profile

Lipids were taken from the lower phase of a Fölch extraction, which was dried under a nitrogen flow and methylated with toluene and sodium methoxide (Sigma, USA) at room temperature for 24 hours. Then, fatty acid methyl esters were dissolved in 50% hexane and recovered from the hexane phase to be dried in nitrogen and suspended in hexane. Separation was achieved in a Supelco fused silica capillary column (30 m × 0.25 mm × 0.25 *μ*m), with a 20 cm/s nitrogen flow rate (mobile phase) and 2°C/min gradient. A Perkin Elmer 500 CLARUS GLC chromatograph with flame ionization detection (Waltham, USA) was used for analysis (oven program: 180°C–240°C). The standard was from NU-Chek-Prep Inc. (USA) and results were expressed as percentages of total fatty acid content.

### 2.6. Statistical Analysis

Data were expressed as mean ± standard error (SE) from at least three separate experiments performed in triplicate, unless otherwise noted. ANOVA models were used to evaluate differences between treatments, followed by Tukey's test for mean comparisons. Then, GLM were suited to regress the effects of experimental conditions (*P* < 0.05). Analyses were performed with the InfoStat 2012 software (InfoStat Group, Argentina).

## 3. Results

### 3.1. Bioguided Extract Selection

#### 3.1.1. Extraction

**Figure 1:**
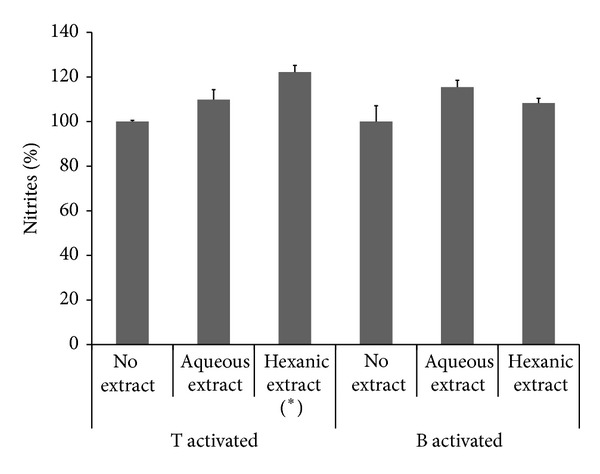
Nitrite % in T- and B-activated cultures treated for 2 h with 200 *μ*g/mL of* L. grisebachii *extracts. Results were averaged from three separate experiments (_ _*P < 0.01).

**Figure 2:**
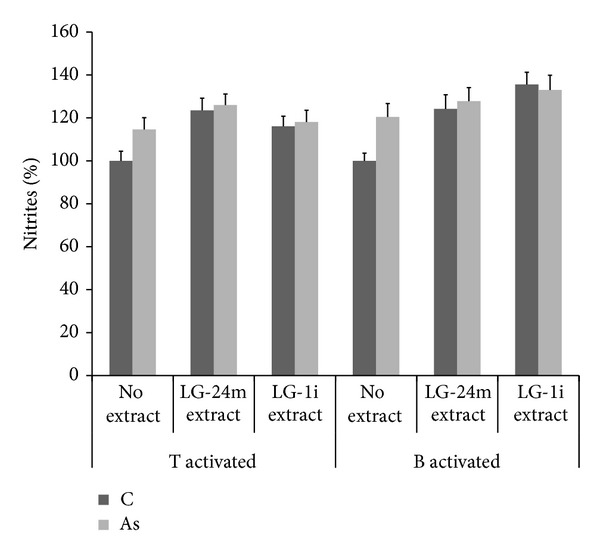
Nitrite % in T- and B-activated cultures from control (C) and arsenic-exposed rats (As), treated for 72 h with 100 *μ*g/mL of aqueous* L. grisebachii* extracts (LG-24m: cold 24 h maceration versus LG-1i: hot 1 h infusion) or without them. Results were averaged from three separate experiments (_ _*P < 0.01).

Given that solvent polarity determines the type of extracted compounds, hexane and water were compared. Hexanic* L. grisebachii *extraction produced an organic fraction that induced nitrite formation in T-activated cultures during the acute assay (*P* < 0.0001). On the other hand, water extraction released an aqueous fraction without nitrite inducing activity in either lymphocyte type. Thus, these polar derivatives were also safe for B- and T-activated cells, which came from* in vivo* As-exposed animals; that is, their safety was an exposure-independent effect and found in proliferated and proliferating cells (Figures 1 and 2). Therefore, aqueous extracts were selected for the next stage.

#### 3.1.2. Redox Efficiency

**Figure 3:**
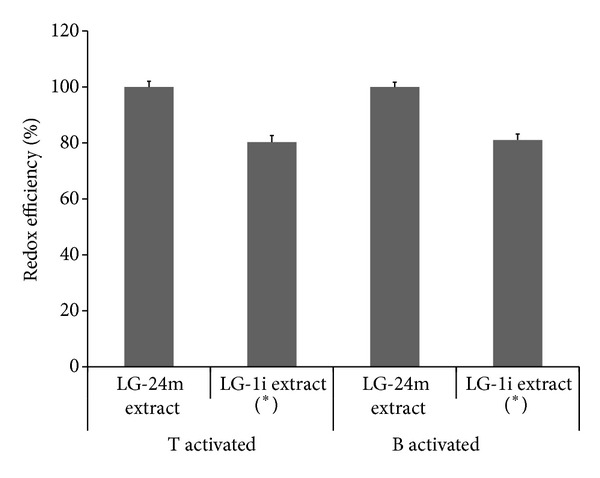
Redox efficiency (cell free radical level/extract phenolic content) of 100 *μ*g/mL aqueous* L. grisebachii* extracts (LG-24m: cold 24 h maceration versus LG-1i: hot 1 h infusion) in T- and B-activated cultures treated for 72 h. Results were averaged from three separate experiments (_ _*P < 0.01).

Given that higher temperature promotes molecular mobility, caloric changes of water could modify the extraction profile. The 24 h water-macerated extract demonstrated higher quotients than the infusive extract (*P* < 0.01), without arsenic-related differences; that is, the last extract showed a better antioxidant capacity per phenolic amount in all cell cultures (Figure 3). Thus, the infusive fraction was selected.

#### 3.1.3. Protection against Arsenotoxicity

**Figure 4:**
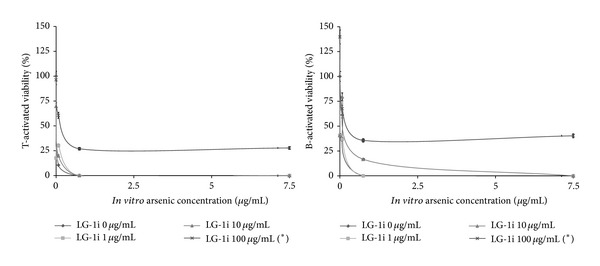
Viability of T- and B-activated cells treated for 72 h with 0–100 *μ*g/mL of the 1 h infusion* L. grisebachii* extract (LG-1i) and 0–7.5 *μ*g/mL of arsenic. Percentages with respect to control (0 *μ*g/mL LG-1i, 0 *μ*g/mL As) were average from four separate experiments (_ _*P < 0.0001).

Given that* in vivo* As exposure has been related to a decrease in splenocyte viability, an* in vitro* assaying of* L. grisebachii *to explore its potential to combat this toxin was encouraged. First, arsenic dose-dependent toxicity was confirmed (concentrations as low as 0.075 *μ*g/mL) (*P* < 0.05). In this case, the infusive extract counteracted such toxicity in a dose-dependent manner (Figure 4) with 100 *μ*g/mL reducing cell death at all As concentrations (including 7.5 *μ*g/mL) (*P* < 0.0001). The dose of 10 *μ*g/mL was protective up to 0.075 *μ*g/mL of As in both cell types. On the other hand, B-activated splenocytes were protected up to 0.75 *μ*g/mL of As, indicating increased resistance. Lower extract concentrations were not sufficient to prevent As-induced damage related to oxidant induction (*P* < 0.05). T-activated cells were more liable than B-activated ones (111.24 ± 0.40% versus 100.00 ± 0.73%, resp.) (*P* < 0.005), whereas the infusive extract was antioxidant in both (*P* < 0.01).

### 3.2. Phytochemistry of the Selected Infusive* Lantana grisebachii* Extract

#### 3.2.1. Phenolics

**Figure 5:**
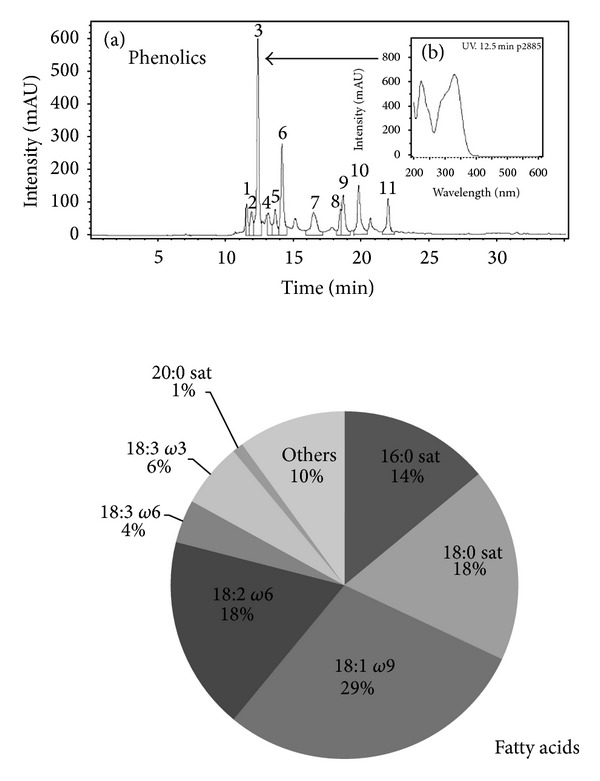
Chromatographic analysis of the infusive* Lantana grisebachii* extract: phenolics with a 0.723 mg/g chlorogenic (*arrow*) and fatty acids (%; others-each one <1%—: 14 : 0, 14 : 1 *ω*9, 16 : 1 *ω*7, 20 : 1 *ω*9, 20 : 3 *ω*3, 20 : 4 *ω*6, 22 : 1 *ω*9, 22 : 5 *ω*3, 24 : 0, and 24 : 1 *ω*9) (_ _*P < 0.05).

It is known that solvent polarity and temperature determine extraction outcome. The employment of water yielded 1.67 times more phenolic extraction from LG than hexane (distinct polarities) after 24 h maceration at room temperature (*P* < 0.05). Also, water extraction could be reduced to 1 hour by increasing its temperature. This method increased phenolic extraction 1.55 times over the classic 24 h water maceration, with extraction being temperature dependent (*P* < 0.02). This infusive phytoextract contained 10.23 ± 0.43 mg/g of phenolics, with 58.46% of flavonoids (05.98 ± 0.12 mg/g). Among phenolics, chlorogenic acid was the predominant compound (0.723 mg/g), among 10 unknown minor compounds (Figure 5).

#### 3.2.2. Lipids

Increased water temperature allows some organic compounds to be extracted; thus, a fatty acid fingerprint could be assessed. The result showed one-third of saturated fatty acids, one-third of *ω*9, *ω*6 (~24%), and *ω*3 (~4%), and scarce *ω*7 (Figure 5).

## 4. Discussion

This study pursued the bioguided identification of a plant extract of* L. grisebachii *(LG) that could combat As lymphotoxicity by comparing different extraction methods and lymphocyte responses (nitrites, free radicals, and cellular viability).

The phenolic increase found in aqueous LG extracts was caused by the presence of principal bioactive molecules, such as flavonoids and phenolic acids (chlorogenic). This was enhanced by the use of heated water, thus improving extraction [21]. Although hydrophilic organic solvents (e.g., ethanol) are usually utilized to obtain these kinds of compounds [22], a pharmacological equivalency has been demonstrated between alcoholic extracts and those derived from infusions [23]. Therefore, flavonoids become bioavailable in humans due to the presence of functional chemical groups (e.g., hydrophilic hydroxyl and carbonyl) in their polycyclic structures [24]. Moreover, the better redox efficiency of infusions with respect to the other cold water-macerated extracts indicated qualitative differences with greater bioactivity per weight. This might be related to extraction of hydrosoluble thermostable antioxidants from plants [25]. Furthermore, organic extracts of other plants have been reported as antioxidants and cytoprotectors but in a lesser extent than their aqueous counterparts [26]. In this study, an elevation of nitrites was seen in cell cultures treated with the hexanic extract, which correlated with phenolic decrease. Concerning this, LG metabolome might present apolar oxidants (e.g., nitrosative inducers, oxygenated fatty acids) [27, 28].

Given that lymphocyte response can be affected by different factors (e.g., cell cycle progression, environmental exposure, etc.), the safety of aqueous extracts was reevaluated. Proliferating lymphocytes were more susceptible to oxidative stress induced by As, as was expected [29]. Nevertheless, aqueous extracts were safe in all lymphocyte cultures. Also, the infusive antioxidant phytoextract of* Lantana grisebachii* triggered a xenohormetic defence against arsenic lymphotoxicity. Some of the pathways involved in such protection have been established [30]. Also, differences between T- and B-activated splenocytes have shown higher B resistance to chemical/environmental stress related to their reduced biologically conditioned susceptibility [31, 32]. Furthermore, the extract may contain B lymphoproliferative compounds such as other plant phenolic derivatives [33]. On other hand, the involvement of apoptosis as the primary lymphotoxic effect has been demonstrated* in extenso* under the assayed conditions [34]; thus, lethal phenotype was not searched (in fact, late determinations in cell culture end could lead to confusions about the initial type). Therefore, a cell-based method for high throughput screenings of phytodrugs was selected [35] due to its representativeness of immune cell response and apoptosis [36].

These multiple effects (antioxidation, cytoprotection, and functional induction) have been seen in other* in vitro* systems. For example, 100 *μ*g/mL LG-1i promoted kidney cells of* Cercopithecus aethiops* (viability with respect to control: 104.67 ± 0.05%) with decreased *γ*-glutamyl transpeptidase activity after being treated for 2 h (control: 27.95 ± 1.55 versus LG-1i: 22.50 ± 0.51 nIU/cell, *P* < 0.05) and 4 h (control: 31.60 ± 0.64 versus LG-1i: 23.15 ± 0.64 nIU/cell, *P* < 0.05) (*unpublished data*). This cell type was the first one where cytoprotective LG was discovered to prevent As-induced oxidative stress [11], with the enzyme being a cell response to augment redox resistance [37]. This supported that phytodrugs (e.g., flavonoid-related compounds, flavolignanes) can stimulate molecular protective pathways [38]. Also, the beneficial effects of* Lantana grisebachii* infusion in both murine lymphocytes and monkey renocytes indicated that they were independent of species and cell types; that is, a general antioxidant bioactivity was found for this plant.

Given that temperature favours lipid kinetics in aqueous biological samples [39], solvent heating was mandatory to extract them from plant material during water extraction, with fatty acid assessment in a plant infusion being innovative. In fact, the extraction enhancement achieved by heating water was manifested by the presence of lipids in an aqueous infusion. This methodological approach favoured unsaturated fatty acids obtaining, including some essential ones for immunological responses, which are highly sensitive to arsenic-induced dysfunctions and disturbances [30].

## 5. Conclusion

Although further studies are required in order to establish other functional implications for lymphocytes, this study provides, for the first time, the basis to develop* Lantana grisebachii*-derived phytodrugs to reduce dysfunctions induced by arsenic, a well-known oxidative immunotoxic. In this regard, arsenicosis is a public health concern worldwide, despite efforts to remedy contaminated soil and water, with immune cells being major targets. Accordingly, sequential bioguided bioprospecting of antioxidant plants, such as* Lantana grisebachii*, is a valuable approach. Moreover, the mentioned plant is immunoactive and common in Argentine flora, which might represent an abundant source of compounds for phytopharmaceutical development by easy extraction using a water-based photoprotected thermoassisted method.

## References

[1] Goleniowski ME, Bongiovanni GA, Palacio L, Nuñez CO, Cantero JJ (2006). Medicinal plants from the “Sierra de Comechingones”, Argentina. *Journal of Ethnopharmacology*.

[2] Surh Y-J (2011). Xenohormesis mechanisms underlying chemopreventive effects of some dietary phytochemicals. *Annals of the New York Academy of Sciences*.

[3] Bongiovanni GA, Soria EA, Eynard AR (2007). Effects of the plant flavonoids silymarin and quercetin on arsenite-induced oxidative stress in CHO-K1 cells. *Food and Chemical Toxicology*.

[4] Nasreddine L, Parent-Massin D (2002). Food contamination by metals and pesticides in the European Union. Should we worry?. *Toxicology Letters*.

[5] Janeway CA, Travers P, Walport M, Shlomchik MJ (2001). *Immunobiology: The Immune System in Health and Disease*.

[6] Singh MK, Yadav SS, Gupta V, Khattri S (2013). Immunomodulatory role of *Emblica officinalis* in arsenic induced oxidative damage and apoptosis in thymocytes of mice. *BMC Complementary and Alternative Medicine*.

[7] Soria EA, Eynard AR, Bongiovanni GA (2010). Cytoprotective effects of silymarin on epithelial cells against arsenic-induced apoptosis in contrast with quercetin cytotoxicity. *Life Sciences*.

[8] Colognato R, Coppedè F, Ponti J, Sabbioni E, Migliore L (2007). Genotoxicity induced by arsenic compounds in peripheral human lymphocytes analysed by cytokinesis-block micronucleus assay. *Mutagenesis*.

[9] Ramsey KA, Foong RE, Sly PD, Larcombe AN, Zosky GR (2013). Early life arsenic exposure and acute and long-term responses to influenza a infection in mice. *Environmental Health Perspectives*.

[10] Barboza GE, Cantero JJ, Núñez C, Pacciaroni A, Ariza Espinar L (2009). Medicinal plants: a general review and a phytochemical and ethnopharmacological screening of the native Argentine Flora. *Kurtziana*.

[11] Soria EA, Goleniowski ME, Cantero JJ, Bongiovanni GA (2008). Antioxidant activity of different extracts of Argentinian medicinal plants against arsenic-induced toxicity in renal cells. *Human and Experimental Toxicology*.

[12] Borneo R, León AE, Aguirre A, Ribotta P, Cantero JJ (2009). Antioxidant capacity of medicinal plants from the Province of Córdoba (Argentina) and their in vitro testing in a model food system. *Food Chemistry*.

[13] Tsao R (2010). Chemistry and biochemistry of dietary polyphenols. *Nutrients*.

[14] Cabrera AL (1976). Regiones fitogeográficas argentinas. *Enciclopedia Argentina de Agricultura Y Jardinería*.

[15] Rubatto Birri PN, Pérez RD, Cremonezzi D, Pérez CA, Rubio M, Bongiovanni GA (2010). Association between As and Cu renal cortex accumulation and physiological and histological alterations after chronic arsenic intake. *Environmental Research*.

[16] Sinha D, Dey S, Bhattacharya RK, Roy M (2007). In vitro mitigation of arsenic toxicity by tea polyphenols in human lymphocytes. *Journal of Environmental Pathology, Toxicology and Oncology*.

[17] Strotmann UJ, Butz B, Bias W-R (1993). The dehydrogenase assay with resazurin: practical performance as a monitoring system and Ph-dependent toxicity of phenolic compounds. *Ecotoxicology and Environmental Safety*.

[18] Green LC, Wagner DA, Glogowski J, Skipper PL, Wishnok JS, Tannenbaum SR (1982). Analysis of nitrate, nitrite, and [15N]nitrate in biological fluids. *Analytical Biochemistry*.

[19] Ait Baddi G, Cegarra J, Merlina G, Revel JC, Hafidi M (2009). Qualitative and quantitative evolution of polyphenolic compounds during composting of an olive-mill waste-wheat straw mixture. *Journal of Hazardous Materials*.

[20] Grosso GS, Carvajal IVC, Principal J (2007). Perfil de flavonoides e índices de oxidación de algunos propóleos colombianos. *Zootecnia Tropical*.

[21] Vuong QV, Golding JB, Stathopoulos CE, Nguyen MH, Roach PD (2011). Optimizing conditions for the extraction of catechins from green tea using hot water. *Journal of Separation Science*.

[22] Abbasi MA, Rubab K, Riaz T, Shahzadi T, Khalid M, Ajaib M (2012). In vitro assessment of relief to oxidative stress by different fractions of *Boerhavia procumbens*. *Pakistan Journal of Pharmaceutical Sciences*.

[23] Ito T, Kakino M, Tazawa S (2012). Quantification of polyphenols and pharmacological analysis of water and ethanol-based extracts of cultivated agarwood leaves. *Journal of Nutritional Science and Vitaminology*.

[24] Beevi SS, Mangamoori LN, Gowda BB (2012). Polyphenolics profile and antioxidant properties of *Raphanus sativus L.*. *Natural Product Research*.

[25] Jäger S, Beffert M, Hoppe K, Nadberezny D, Frank B, Scheffler A (2011). Preparation of herbal tea as infusion or by maceration at room temperature using mistletoe tea as an example. *Scientia Pharmaceutica*.

[26] Tripathi YB, Chaturvedi AP, Pandey N (2012). Effect of nigella sativa seeds extracts on inos through antioxidant potential only: crude/total extract as molecular therapy drug. *Indian Journal of Experimental Biology*.

[27] Morihara N, Sumioka I, Ide N, Moriguchi T, Uda N, Kyo E (2006). Aged garlic extract maintains cardiovascular homeostasis in mice and rats. *Journal of Nutrition*.

[28] Doehlert DC, Rayas-Duarte P, McMullen MS (2011). Inhibition of fusarium graminearum growth in flour gel cultures by hexane-soluble compounds from oat (*Avena sativa L.*) flour. *Journal of Food Protection*.

[29] Thomas-Schoemann A, Batteux F, Mongaret C (2012). Arsenic trioxide exerts antitumor activity through regulatory T cell depletion mediated by oxidative stress in a murine model of colon cancer. *Journal of Immunology*.

[30] Ramos Elizagaray SI (2013). *Actividad quimiopreventiva e inmunoprotectora del extracto acuoso de Lantana grisebachii var. grisebachii en hidroarsenicismo experimental [M.S. thesis]*.

[31] Turner JE, Bosch JA, Aldred S (2011). Measurement of exercise-induced oxidative stress in lymphocytes. *Biochemical Society Transactions*.

[32] Shen C-C, Liang H-J, Wang C-C, Liao M-H, Jan T-R (2011). A role of cellular glutathione in the differential effects of iron oxide nanoparticles on antigen-specific T cell cytokine expression. *International Journal of Nanomedicine*.

[33] Francus T (1994). Plant polyphenolic-protein conjugates activate murine spleen cells and bind to multiple cell surface components. *Proceedings of the Society for Experimental Biology and Medicine*.

[34] Yu H-S, Liao W-T, Chang K-L, Yu C-L, Chen G-S (2002). Arsenic induces tumor necrosis factor *α* release and tumor necrosis factor receptor 1 signaling in T helper cell apoptosis. *Journal of Investigative Dermatology*.

[35] O'Neill TE, Li H, Colquhoun CD, Johnson JA, Webster D, Gray CA (2014). Optimisation of the microplate resazurin assay for screening and bioassay-guided fractionation of phytochemical extracts against mycobacterium tuberculosis. *Phytochemical Analysis*.

[36] Zhi-Jun Y, Sriranganathan N, Vaught T, Arastu SK, Ahmed SA (1997). A dye-based lymphocyte proliferation assay that permits multiple immunological analyses: mRNA, cytogenetic, apoptosis, and immunophenotyping studies. *Journal of Immunological Methods*.

[37] Quiroga A, Quiroga PL, Martínez E, Soria EA, Valentich MA (2010). Anti-breast cancer activity of curcumin on the human oxidation-resistant cells ZR-75-1 with *γ*-glutamyltranspeptidase inhibition. *Journal of Experimental Therapeutics and Oncology*.

[38] Sonnenbichler J, Scalera F, Sonnenbichler I, Weyhenmeyer R (1999). Stimulatory effects of silibinin and silicristin from the milk thistle *Silybum marianum* on kidney cells. *Journal of Pharmacology and Experimental Therapeutics*.

[39] Milhaud J (2004). New insights into water-phospholipid model membrane interactions. *Biochimica et Biophysica Acta—Biomembranes*.

